# Cost‐effectiveness of second‐line ipilimumab for metastatic melanoma: A real‐world population‐based cohort study of resource utilization

**DOI:** 10.1002/cam4.5862

**Published:** 2023-03-31

**Authors:** Brandon Lu, Wei Fang Dai, Ruth Croxford, Wanrudee Isaranuwatchai, Jaclyn Beca, Ines B. Menjak, Teresa M. Petrella, Nicole Mittmann, Craig C. Earle, Scott Gavura, Rebecca E. Mercer, Timothy P. Hanna, Kelvin K. W. Chan

**Affiliations:** ^1^ Sunnybrook Health Sciences Centre Toronto Ontario Canada; ^2^ Canadian Centre for Applied Research in Cancer Control Toronto Ontario Canada; ^3^ Ontario Health (Cancer Care Ontario) Toronto Ontario Canada; ^4^ ICES Toronto Ontario Canada; ^5^ St Michael's Hospital Toronto Ontario Canada; ^6^ Institute of Health Policy, Management and Evaluation University of Toronto Toronto Ontario Canada; ^7^ Department of Medicine University of Toronto Toronto Ontario Canada; ^8^ University of Toronto Toronto Ontario Canada; ^9^ Canadian Agency for Drugs and Technologies in Health Ottawa Ontario Canada; ^10^ Division of Cancer Care and Epidemiology Queen's University Cancer Research Institute Kingston Ontario Canada; ^11^ Department of Oncology Queen's University Kingston Ontario Canada; ^12^ ICES, Queen's University Kingston Ontario Canada

**Keywords:** cost‐effectiveness, ipilimumab, metastatic melanoma, oncology, real‐world evidence

## Abstract

**Background:**

The efficacy‐effectiveness gap between randomized trial and real‐world evidence regarding the clinical benefit of ipilimumab for metastatic melanoma (MM) has been well characterized by previous literature, consistent with initial concerns raised by health technology assessment agencies (HTAs). As these differences can significantly impact cost‐effectiveness, it is critical to assess the real‐world cost‐effectiveness of second‐line ipilimumab versus non‐ipilimumab treatments for MM.

**Methods:**

This was a population‐based retrospective cohort study of patients who received second‐line non‐ipilimumab therapies between 2008 and 2012 versus ipilimumab treatment between 2012 and 2015 (after public reimbursement) for MM in Ontario. Using a 5‐year time horizon, censor‐adjusted and discounted (1.5%) costs (from the public payer's perspective in Canadian dollars) and effectiveness were used to calculate incremental cost‐effectiveness ratios (ICERs) in life‐years gained (LYGs) and quality‐adjusted life years (QALYs), with bootstrapping to capture uncertainty. Varying the discount rate and reducing the price of ipilimumab were done as sensitivity analyses.

**Results:**

In total, 329 MM were identified (Treated: 189; Controls: 140). Ipilimumab was associated with an incremental effectiveness of 0.59 LYG, incremental cost of $91,233, and ICER of $153,778/LYG. ICERs were not sensitive to discounting rate. Adjusting for quality of life using utility weights resulted in an ICER of $225,885/QALY, confirming the original HTA estimate prior to public reimbursement. Reducing the price of ipilimumab by 100% resulted in an ICER of $111,728/QALY.

**Conclusion:**

Despite its clinical benefit, ipilimumab as second‐line monotherapy for MM patients is not cost‐effective in the real world as projected by HTA under conventional willingness‐to‐pay thresholds.

## Abstract 2

In light of concerns regarding the diminished survival benefit of second‐line ipilimumab for metastatic melanoma in the real world, this population‐based cost‐effectiveness analysis validates trial based model estimations by the Canadian Agency for Drugs and Technologies in Health to conclude that ipilimumab is not cost‐effective compared to non‐ipilimumab treatments. The findings of this study highlights the value of incorporating real‐world evidence to inform life‐cycle reassessment.

## 1 INTRODUCTION

Ipilimumab was the first immunotherapy treatment approved by the U.S. Food & Drug Administration and European Medicines Agency that had demonstrated significant survival benefit beyond first‐line therapy for patients with recurrent or metastatic melanoma (MM) from phase 3 trial evidence.^1^ Before the introduction of ipilimumab, patients with MM were limited to systemic therapies such as dacarbazine and temozolomide,^2^, ^3^, ^4^ which conferred limited benefit and only modest response rates. With a median overall survival (OS) of 6 to 9 months and <5% survival rate at 5 years,^4^ the prognosis for patients with MM remained poor.

Despite demonstrating substantial clinical benefit, ipilimumab was deemed not cost‐effective at the initial health technology assessment (HTA) review.^5^ According to the manufacturer's model submitted to the Canadian Agency for Drugs and Technologies in Health (CADTH),^6^ the incremental cost of ipilimumab compared to other second‐line chemotherapies ranged between $70,247 and $118,942. The incremental clinical benefit of ipilimumab, as measured by quality‐adjusted life‐years (QALYs), ranged from 0.676 to 0.749 QALYs, and the estimated incremental cost‐effectiveness ratio (ICER) ranged between $103,839/QALY and $166,186/QALY. A re‐analysis of the manufacturer's model conducted by the economic guidance panel at CADTH over a five‐year time horizon estimated the ICER to be $269,299/QALY.^6^ Given the clinical benefit of second‐line ipilimumab, CADTH recommended for the public funding of ipilimumab conditional upon pricing arrangements to improve cost‐effectiveness. On September 13, 2012, ipilimumab became the first immunotherapy indication to receive regulatory approval and public funding in Canada.

In the manufacturer's submission to both CADTH and the U.K. National Institute for Health and Care Excellence (NICE), survival benefit was determined using the MDX010‐20 trial.^1^ With <5 years of follow‐up data and <3 years of median follow‐up, NICE expressed concern regarding the limited information available that could be used to infer the cost of treatment and outcome beyond the trial horizon.^7^ Previous literature has demonstrated that the median OS for patients receiving second‐line ipilimumab was less than that observed in the trial.^8^ In particular, while the real‐world survival probability of second‐line ipilimumab at 2 years was similar to that observed in the trial (real world: 21%; trial: 25%), the survival probability at 3 years was half of that observed in the trial (real world: 14.3%; trial: 25%).^9^ This difference illustrates that extrapolation of survival curves for model estimation is not always valid. In addition, patients treated with second‐line ipilimumab had more adverse events that resulted in hospital visits in the real world than what was observed in the trial.^10^ This decrease in survival benefit and increase in adverse events associated with second‐line ipilimumab can significantly impact the cost‐effectiveness estimate.^9^, ^10^

To date, there are no published studies evaluating the real‐world cost‐effectiveness of second‐line ipilimumab for patients with MM to verify the original estimates projected by HTAs and to confirm value‐for‐money in the routinely treated, unselected population. While the therapeutic landscape for MM has evolved since the introduction of ipilimumab and other novel therapies, MM remains a valuable platform for demonstrating the feasibility of generating real‐world evidence (RWE) through population‐level analyses to create opportunities for health technology reassessment. Thus, we sought to examine the real‐world cost‐effectiveness of second‐line ipilimumab compared to historical controls in patients with MM to validate the original economic analysis from CADTH.

## 2 METHODS

### 2.1 Study design and cohort creation

This was a real‐world population‐based retrospective cohort analysis conducted in Ontario, the most populous province with a population nearing 15 million.^11^ In Ontario, public drug funding programs for cancer drugs under a single government‐funded health insurance system typically reimburses most cancer care administered in hospitals and cancer clinics. Through the New Drug Funding Program (NDFP) administered by Ontario Health (formerly Cancer Care Ontario (CCO)), ipilimumab as second‐line monotherapy for MM became publicly funded in Ontario on September 13, 2012. This study conforms to the reporting standards outlined by the RECORD‐PE, CHEERS, and STaRT‐RWE checklists.^12^, ^13^, ^14^

Using the Ontario Cancer Registry, adult patients (≥18 years old) with an incident diagnosis of melanoma (International Classification of Disease for Oncology: diagnosis code C44) were identified. This cohort was linked to Ontario Health (CCO)'s Activity Level Reporting (ALR) systemic database to identify the initiation of the first‐line treatment regimen for advanced melanoma on or after April 1, 2005. The ALR systemic database records chemotherapy and supportive treatments provided to patients in hospital chemotherapy clinics during each treatment visit. First‐line treatment regimens included non‐interferon systemic therapy with palliative intent. The study cohort was further linked to the NDFP and ALR databases to ascertain second‐line treatments, based on a change in systemic therapy agents after first‐line treatment.

Historical controls included patients that received second‐line therapy between September 13, 2008, and September 13, 2012. Patients that started second‐line ipilimumab treatment after public funding between September 13, 2012, and March 31, 2015, were included as cases. Beyond this date, first‐line ipilimumab became publicly funded for MM, thus cohort accrual was ended to prevent misclassification. The index date of treatment was defined as the first dose of second‐line treatment. The study cohort was followed up until March 31, 2020, or until death if before March 31, 2020. We excluded patients that had other cancer diagnoses, received ipilimumab before public funding (September 13, 2012), received first‐line ipilimumab, received ipilimumab with another drug, or received second‐line clinical trial agents (Appendix A).

### 2.2 Baseline covariates and data sources

We linked the study cohort to several health administrative databases to ascertain baseline characteristics (Appendix B). These datasets were linked using unique, encoded identifiers and analyzed at ICES, an independent, non‐profit research institute funded by an annual grant from the Ontario Ministry of Health and Long‐Term Care.^15^ Under Ontario's privacy legislation, ICES is authorized to collect and use health care data for the purposes of health system analysis, evaluation, and decision support. Secure access to these data is governed by policies and procedures that are approved by the Information and Privacy Commissioner of Ontario. Small cell counts (<6 patients) were suppressed to mitigate any risk of re‐identification. Research ethics clearance was granted through the Sunnybrook Research Ethics Board.

### 2.3 Statistical analysis

We calculated descriptive statistics for the baseline variables, using frequencies and percentages for categorical variables, and mean and standard deviation (SD) or median and interquartile ranges for continuous variables. Chi‐square tests, Wilcoxon signed‐ranked tests, and analysis of variance were used to calculate differences between groups. A logistic regression model based on measures of patient comorbidity measured by the Charlson Comorbidity Index and ACG (The John Hopkins ACG® System, version 10.0), age, place of residence, time from diagnosis to start of second‐line therapy, and prior radiation treatment were used to calculate propensity scores. We adjusted for differences between groups using inverse probability of treatment weighting (IPTW). For all baseline variables, we calculated weighted standardized differences, with adequate balance between groups represented by a standardized difference of <0.1.^16^ All analyses were conducted in SAS 9.4 (SAS Institute Inc., Cary, NC, USA).

### 2.4 Treatment effectiveness

We measured treatment effectiveness in life years (LY) and QALY. LY was defined as the 5‐year survival time from the initiation of second‐line therapy to the date of death or censoring. Patients were censored at date of death, the date of maximum follow up, administrative censoring, or 5 years after the initiation of second‐line therapy. The number of patients with complete costs after 1500 days were few, since the survival curves had flattened beyond 52 months with survival rates <15% in both groups (Appendix C).

QALY was obtained by adjusting LY with utility weights from the Canadian general public used in the initial CADTH drug review.^17^ Each patient's survival time was divided into two health states, progression‐free and progressed. The progression‐free state was defined as the time on second‐line treatment or end of second‐line treatment to 6 months before death without third‐line treatment. The progressed state was defined as the start of third‐line treatment or 6 months within death after end of second‐line treatment. Patients with survival <6 months were assigned to the worse health state only after the end of their second‐line treatment. Utility weights for the progression‐free state were sampled from a beta distribution with a mean of 0.79 and an SD of 0.02. The utility for the progressed state was subtracted from the sampled utility from first health state as a disutility with a mean of 0.24 and SD of 0.02. In each bootstrap iteration, utility values were sampled once per person.

### 2.5 Cost analysis

The 5‐year total patient level costs of publicly funded healthcare services were calculated using linked administrative databases (Appendix D), including acute inpatient hospitalizations, emergency department visits, outpatient visits, homecare visits, same‐day surgeries, long‐term care, continuing care, medications from the Ontario Drug Benefit Program (ODBP), and medications from NDFP (Appendix B). Costs were calculated from the public payer's perspective using standardized methods based on costing macros at ICES.^18^, ^19^

### 2.6 Cost‐effectiveness analysis

Inverse Probability of Censoring Weighting (IPCW) nonparametric methods were used to adjust the total LY, QALY, and costs for censoring.^20^ Censoring adjustment was conducted by dividing study follow‐up into intervals of 30 days from index date. The probability of not being censored at the beginning of each interval for each treatment group was generated based on Kaplan–Meier estimates. The total LY and costs in each interval for each patient were divided by the probability of not being censored at the beginning of the interval. Adjusted life‐years gained (LYG) and costs for each patient were summed across each time interval. Both costs and survival time were discounted at 1.5% annually.^21^

The primary outcome of interest was the incremental cost‐effectiveness ratio (ICER). We divided the difference in mean total cost between groups by the difference in mean LY to calculate the ICER and adjusted for IPTW and administrative censoring. We performed 1000 iterations of bootstraps to calculate the 95% confidence interval to capture the underlying uncertainty in the ICERs. The results of ICERs comparing cases and controls were plotted on the cost‐effectiveness plane.

Incremental net monetary benefit (NMB) was the secondary outcome of interest. For each patient, we calculated the net benefit value using a willingness‐to‐pay (WTP) threshold value, incremental effect, and incremental cost; we then calculated incremental NMB using net benefit regression with treatment group as the predictor.^22^, ^23^ A positive incremental NMB value indicates that ipilimumab is cost‐effective at the specified WTP threshold. Different values of WTP were calculated ranging between $0 to $300,000/LYG.

### 2.7 Sensitivity analysis

Sensitivity analyses were used to evaluate alternative assumptions which included varying the discounting rate from 0% to 3.0% and examining the ICER when the price of ipilimumab was reduced from 0% to 100%.

## 3 RESULTS

### 3.1 Study population and baseline characteristics

We identified 329 MM patients who received second‐line treatments between September 13, 2008, and March 31, 2015, of which 140 (42.6%) were historical controls and 189 (57.4%) were treated with ipilimumab (Appendix A). Apart from age and income quintile, no significant differences in measured characteristics were observed after IPTW adjustment in Table 1 (see Dai et al.^9^ for unweighted sample).

**TABLE 1:** Baseline cohort characteristics by treatment group for IPTW weighted sample^a^, ^b^

Variables	Historical controls	Second line Ipilimumab	Weighted standardized difference
	N = 288.4	N = 311.2	
Age at second line (years), mean (SD)	57.5 (18.5)	59.5 (17.9)	0.11
Males, n (%)	66.63%	67.71%	0.02
Income quintile, n (%)			
Lowest	11.47%	10.39%	0.03
Medium to Low	15.73%	16.47%	0.02
Medium	26.00%	16.99%	0.22
Medium to High	19.88%	26.89%	0.17
Highest	26.93%	29.26%	0.05
Urban, n (%)	88.53%	89.12%	0.02
Morbidity score, mean (SD)	15.99 (16.8)	16.1 (15.4)	0.01
Number of ADGs, median (IQR)	8.3 (4.5)	8.5 (4.4)	0.04
Charlson's score, n (%)			
0	11.86%	10.82%	0.03
1+	1.95%	3.19%	0.08
Prior radiation (any), n (%)	55.40%	56.00%	0.01
Prior radiation of brain, n (%)	22.37%	22.98%	0.01
Prior radiation other than brain, n (%)	45.04%	40.20%	0.1
Prior resection (any), n (%)	55.40%	56.00%	0.01
Prior brain resection, n (%)	4.99%	6.63%	0.07
Prior other resection, n (%)	6.69%	6.68%	0
First‐line treatment, n (%)			
Chemotherapy	81.67%	58.39%	0.53
BRAF/MEK	9.66%	36.85%	0.68
Others	8.67%	4.75%	0.16
Time from diagnosis to start of second line (months), median (IQR)	23.1 (10.9–47.8)	20.2 (10.1–42.2)	0.04
Time from end of first‐line treatment to start of second‐line treatment (months), median (IQR)	1.5	1.0	0.19
Time from diagnosis to start of first line (months), median (IQR)	13.9	14.9	0.03

Abbreviations: ADG, Adjusted Diagnosis Groups; IQR, Interquartile range; SD, Standard Deviation.

^a^ Adapted from Dai et al.^9^

^b^ Percentages shown in the table are column percentages.

### 3.2 Incremental effectiveness

The 5‐year mean censor‐adjusted treatment effectiveness for patients treated with ipilimumab after applying a 1.5% discount was 1.29 years, as compared to 0.70 years in the control group (Table 2). Second‐line ipilimumab was associated with an incremental effectiveness of 0.59 (95% CI: 0.27–0.89) years at 5 years after initiating treatment. An incremental estimate of 0.40 QALYs was obtained after adjusting survival for quality of life.

**TABLE 2:** Incremental cost‐effectiveness of second‐line ipilimumab vs historical controls in the IPTW‐adjusted cohort, adjusted for censoring using IPCW^a^, ^b^

	Historical controls	Second line ipilimumab	Incremental difference^c^
Mean total cost, $ (95% CI)	65,670	156,903	91,233 (68,014–112,754)
Systemic therapy	20,709	89,494	68,785
Ambulatory hospital care	13,568	23,408	9840
Acute inpatient hospital care	12,190	18,335	6145
Physician claims	7348	11,512	4164
Radiation	3854	5921	2067
Chronic and rehabilitation care	1987	3174	1187
Laboratory tests	120	193	73
Home care services	5972	5225	−747
Mean LYG (95% CI)	0.70	1.29	0.59 (0.27–0.89)
Mean QALY (95% CI)	0.48	0.88	0.40 (0.17–0.64)
ICER (95% CI) ($/LYG)	—	—	153,778 (106,093–300,645)
ICER (95% CI) ($/QALY)	—	—	225,885 (149,876–477,082)

Abbreviations: CI, confidence interval; ICER, incremental cost‐effectiveness ratio; IPCW, inverse probability of censoring weighting; IPTW, inverse probability treatment weighting; LYG, life‐years gained; QALY, quality‐adjusted life years.

^a^ IPCW 5‐year follow‐up.

^b^ Cost and LY discounted at 1.5%, costs expressed as Canadian dollars.

^c^ Incremental difference calculated as second‐line ipilimumab minus historical controls.

### 3.3 Incremental cost

Total costs are broken down by resource category in Table 2. For patients treated with second‐line ipilimumab, the mean 5‐year discounted and censoring‐adjusted costs were $156,903 versus $65,670 for the historical controls. The incremental cost for second‐line ipilimumab was $91,233 (95% CI: 68,014 ‐ 112,754) over 5 years. Cost of systemic therapy was the greatest contributor to the incremental cost differences between the two groups (75%).

### 3.4 Incremental cost‐effectiveness

**FIGURE 1:**
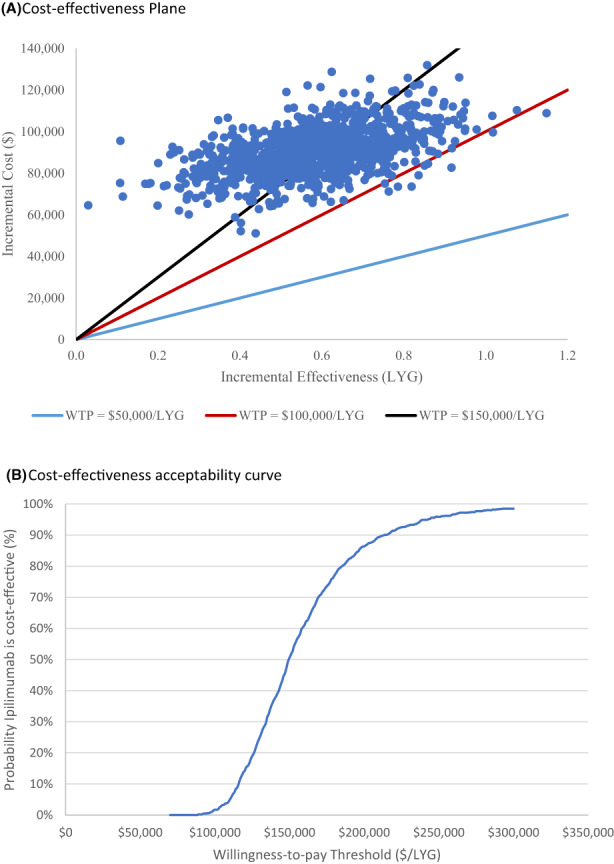
Incremental cost‐effectiveness ratio (A) scatterplot and (B) cost‐effectiveness acceptability curve based on 1000 samples. LYG, life‐year gained; WTP, willingness‐to‐pay.

The ICERs were $153,778/LYG (95% CI: 106,093 ‐ 300,645) and $225,885/QALY (95% CI: 149,876 ‐ 477,082) within a 5‐year time horizon (Table 2). All bootstrapped samples were in the north‐east quadrant of the cost‐effectiveness plane (Figure 1A). Based on list prices, the cost‐effectiveness acceptability curve demonstrated that the probability of second‐line ipilimumab being cost‐effective was 0% if the WTP threshold was considered to be $50,000/LYG (Figure 1B). At a WTP threshold of $100,000/LYG, the probability of cost‐effectiveness was 1.7%.

### 3.5 Incremental net monetary benefit

The estimated incremental NMB values at varying WTP thresholds are presented in Table E1 (Appendix E). At a WTP threshold of $50,000/LYG, $100,000/LYG, and $150,000/LYG, the estimated incremental NMB values were negative. Therefore, ipilimumab is not considered to be cost‐effective at these thresholds based on list prices.

### 3.6 Sensitivity analysis

**FIGURE 2:**
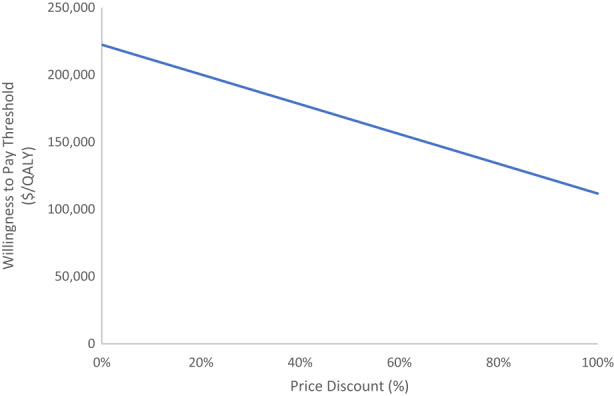
Sensitivity analysis of the price reduction of ipilimumab. QALY, quality‐adjusted life‐year.

The ICERs without discounting and 3.0% discount applied were $151,402/LYG and $156,295/LYG, respectively (Table 3). Results of the price reduction analysis are illustrated in Figure 2. Reducing the price of ipilimumab by 100% resulted in an ICER of $111,728/QALY.

**TABLE 3:** Sensitivity analysis of the incremental cost‐effectiveness of ipilimumab versus historical controls

	Incremental cost ($)		Incremental effectiveness (LYG)		ICER ($/LYG)	
Scenario	Mean	95% CI	Mean	95% CI	Mean	95% CI
Base case	91,233	68,014–112,754	0.59	0.27–0.89	153,778	106,093–300,645
No discounting	92,282	68,603–114,026	0.61	0.27–0.91	151,402	104,268–297,482
3.0% discounting	90,336	67,504–111,511	0.58	0.26–0.86	156,295	107,832–304,661

Abbreviations: CI, confidence interval; ICER, incremental cost‐effectiveness ratio; LYG, life‐years gained.

## 4 DISCUSSION

In this study, we examined the comparative cost‐effectiveness of second‐line ipilimumab to historical controls in patients with MM, using real‐world population‐based data from Ontario, Canada. The present findings show that ipilimumab can only be considered cost‐effective at high WTP thresholds above $150,000/LYG or above $200,000/QALY gained, which is above conventional WTP thresholds. Although we observed a significant survival benefit associated with ipilimumab, the mean total cost was more than twofold that of historical controls ($156,903 versus $65,670) resulting in mean ICERs of $153,778/LYG and $225,885/QALY. The sensitivity analysis demonstrated that variations to the discount rate did not significantly impact the ICER, and that ipilimumab would not be considered cost‐effective at any price.

Our analysis produced results that were broadly in line with CADTH's model‐based incremental cost‐utility ratio estimate of $269,299/QALY,^5^ although estimates from these analyses differed substantially from the estimates provided by the manufacturer for both cost‐effectiveness and clinical benefit. We found that the incremental clinical benefit associated with ipilimumab, as measured in QALY, was 41%–47% lower than that of the manufacturer's estimate.^6^ This decrease in clinical benefit may in part be attributable to patients that were unable to tolerate the standard treatment regimen of four ipilimumab doses, as only half (49.7%) of the patients in the ipilimumab group completed the planned treatment regimen.^9^ Notably, the real‐world incremental cost ($91,233) of ipilimumab fell within the range of what was initially reported by the manufacturer ($70,247–$118,942).^6^ Thus, while incremental costs were well captured from the trial given that most costs are incurred upfront for ipilimumab therapy, extending the time horizon beyond the length of the trial provided better estimates of real‐world clinical benefit and cost‐effectiveness, confirming initial projections from CADTH that ipilimumab is not considered cost‐effective at the list price.

With the introduction of other immunotherapies and combination therapies that have shown significant advantages in survival outcomes, it is worth noting that ipilimumab monotherapy has fallen out of favor in the therapeutic landscape for MM. Current clinical approaches include the anti‐programmed cell death protein 1 (PD‐1) agent nivolumab in combination with ipilimumab,^24^ which continues to demonstrate improvements in OS, progression‐free survival, and overall response rate compared to either nivolumab or ipilimumab alone based on 6.5‐year outcomes of the CheckMate‐067 trial.^25^ Overall, real‐world studies that have examined the effectiveness of ipilimumab and nivolumab combination therapy for MM in routine practice have produced results similar to those reported in pivotal trials.^26^, ^27^, ^28^, ^29^ Although associated with significant toxicity, this regimen was considered cost‐effective compared to ipilimumab monotherapy ($66,750/QALY) based on the manufacturer's submission to CADTH and received recommendation for reimbursement in Canada.^30^, ^31^ While other published cost‐effectiveness analyses have provided lower ICER estimates of £4225/QALY,^32^ $6119 USD/QALY,^33^ and $21,143 USD/QALY,^34^ findings have been consistent in that combination immunotherapy with ipilimumab and nivolumab is a more optimal and cost‐effective option.

The results of this study should be interpreted in light of three potential limitations. First, our results are subject to the inherent risk of potential confounding due to the non‐randomized design of our study. Propensity scores were used in balancing the measured characteristics between our comparisons based on data available, however, unobserved characteristics and residual imbalances may have confounded our results. Second, historical comparisons may have been affected by the changing therapeutic landscape of MM over time. Other systemic treatments may have caused differences in cost and survival between the historical controls and the cases, thus potentially confounding our comparisons.^35^ However, real‐world findings have shown little influence of receiving third‐line treatment on the survival of patients treated with second‐line ipilimumab.^9^ Third, an overestimation of drug costs is plausible, since drivers for price reduction in the real world such as price negotiations, discounts, and rebates, are confidential and not publicly available for consideration in our analysis.^35^ This limitation is not unique to our study, but inherent to almost all economic evaluations in published literature, including the initial ICER estimate reported by CADTH.

To our knowledge, there are no published studies that have used real‐world data to evaluate and quantify the comparative cost‐effectiveness of second‐line ipilimumab for MM, which was the first approved indication for contemporary immunotherapy, to historical controls. Despite the limitations of our study, this real‐world analysis utilized population‐based data to confer greater generalizability to our cost‐effectiveness estimates, which is difficult to accomplish using highly selective individual patient data from randomized controlled trials. Furthermore, we demonstrated how RWE provides advantages over model‐based approaches that require assumptions for estimation and often have lower capacity for incorporating various cost components.

The implications of the present study are twofold. First, resolving the initial concerns and uncertainties around the continuing clinical benefit of ipilimumab beyond the trial horizon deliberated by NICE,^7^ our real‐world cost‐effectiveness analysis offers validation of CADTH's initial economic assessment for ipilimumab.^6^ Second, despite the usual concerns about the lack of generalizability of clinical trial efficacy to real‐world effectiveness,^9^, ^10^ our results suggest that cost‐effectiveness estimates based on modeling using initial clinical trial data and other input are not always underestimations of those derived from real‐world settings. Therefore, this study illustrates the importance of life‐cycle reassessment of cost‐effectiveness based on RWE to verify whether the initial trial‐based predictions translate into real‐world settings,^36^ particularly when costs are high and benefit is uncertain. Furthermore, in cases where the real‐world cost‐effectiveness is underestimated by the initial prediction, these findings may provide valuable evidence to support price renegotiations as well as the capacity for revising existing drug funding decisions.^37^, ^38^

## 5 CONCLUSION

Based on the results of this real‐world population‐based cost‐effectiveness analysis, we concluded that second‐line ipilimumab as monotherapy for patients with MM would not be considered cost‐effective at the list price. The incremental clinical benefit of ipilimumab, which was considerably lower than that observed in the initial trial, was not justified by its costs. However, despite less real‐world absolute survival benefit observed in our analysis compared to what was observed in the pivotal clinical trial, our real‐world cost‐effectiveness estimate broadly remained in agreement with trial‐based model estimations from CADTH. Thus, the potential for significant differences between real‐world and trial‐based findings highlights the value of incorporating the use of RWE to inform life‐cycle reassessment, which in turn creates various opportunities to improve drug funding sustainability.

## AUTHOR CONTRIBUTIONS

**Brandon Lu:** Investigation (equal); writing – original draft (equal); writing – review and editing (lead). **Wei Fang Dai:** Investigation (equal); methodology (equal); writing – original draft (equal); writing – review and editing (supporting). **Ruth Croxford:** Data curation (equal); formal analysis (lead); investigation (equal); methodology (equal); validation (equal); writing – review and editing (supporting). **Wanrudee Isaranuwatchai:** Conceptualization (equal); investigation (equal); methodology (equal); writing – original draft (equal); writing – review and editing (supporting). **Jaclyn Beca:** Investigation (equal); methodology (equal); writing – original draft (equal); writing – review and editing (supporting). **Ines Menjak:** Investigation (equal); writing – original draft (equal); writing ‐ review and editing (supporting). **Teresa Petrella:** Investigation (equal); writing – original draft (equal); writing – review and editing (supporting). **Nicole Mittmann:** Investigation (equal); writing – original draft (equal); writing – review and editing (supporting). **Craig C Earle:** Investigation (equal); writing – original draft (equal); writing – review and editing (supporting). **Scott Gavura:** Investigation (equal); writing – original draft (equal); writing – review and editing (supporting). **Rebecca Mercer:** Investigation (equal); writing – original draft (equal); writing – review and editing (supporting). **Timothy Hanna:** Investigation (equal); writing – original draft (equal); writing – review and editing (supporting). **Kelvin Chan:** Conceptualization (equal); investigation (equal); methodology (equal); writing – original draft (equal); writing – review and editing (equal).

## FUNDING INFORMATION

This project was funded by the CATALYST grant for Health Services and Economic Research in Cancer – (Grant # FRN151290) provided by Canadian Institutes of Health Research to Dr. Kelvin Chan. T.P. Hanna holds a research chair provided by the Ontario Institute for Cancer Research through funding provided by the Government of Ontario (#IA‐035).

## CONFLICT OF INTEREST STATEMENT

The authors declare that they have no competing interests.

## ETHICS STATEMENT

The study has received ethics approval from the Research Ethics Board of Sunnybrook Health Sciences Centre, Ontario, Canada.

## Supporting information


Appendix A–F

## Data Availability

The data that support the findings of this study are available from ICES, but restrictions apply to the availability of these data, which were used under license for the current study, and so are not publicly available. Data are however available from the authors upon reasonable request and with permission of ICES.
