# Supplementary material for: Cost‐effectiveness of second‐line ipilimumab for metastatic melanoma: A real‐world population‐based cohort study of resource utilization
Source: Cancer Med. 2023 Mar 31;12(10):11451–61. doi: 10.1002/cam4.5862 (PMC10242360; doi:10.1002/cam4.5862)
Supplement: Supplementary file 1 — Appendix A–F [file CAM4-12-11451-s001.docx]

**Supplementary Appendix**

**Cost-effectiveness of second-line ipilimumab for metastatic melanoma: A real-world population-based cohort study of resource utilization**

**Authors:** Brandon Lu, Wei Fang Dai, Ruth Croxford, Wanrudee Isaranuwatchai, Jaclyn Beca, Ines B. Menjak, Teresa M. Petrella, Nicole Mittmann, Craig C. Earle, Scott Gavura, Rebecca E. Mercer, Timothy P. Hanna, Kelvin K.W. Chan

**Appendix A:** Cohort creation and study design

**Appendix B:** Supplementary methods for baseline covariates and costs

**Appendix C:** Kaplan-Meier survival estimates adjusted for IPTW

**Appendix D:** Data sources

**Appendix E:** Incremental net monetary benefit

**Appendix F:** CHEERS, RECORD-PE**,** and STaRT-RWE checklists

**Appendix A:** Cohort creation and study design

**
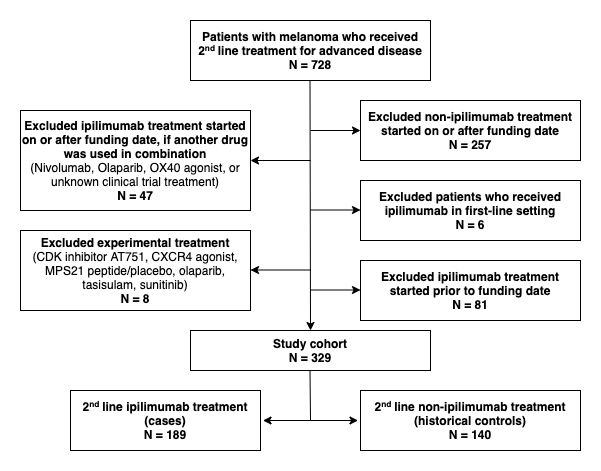
A1.** Cohort creation

**A2.** Study design


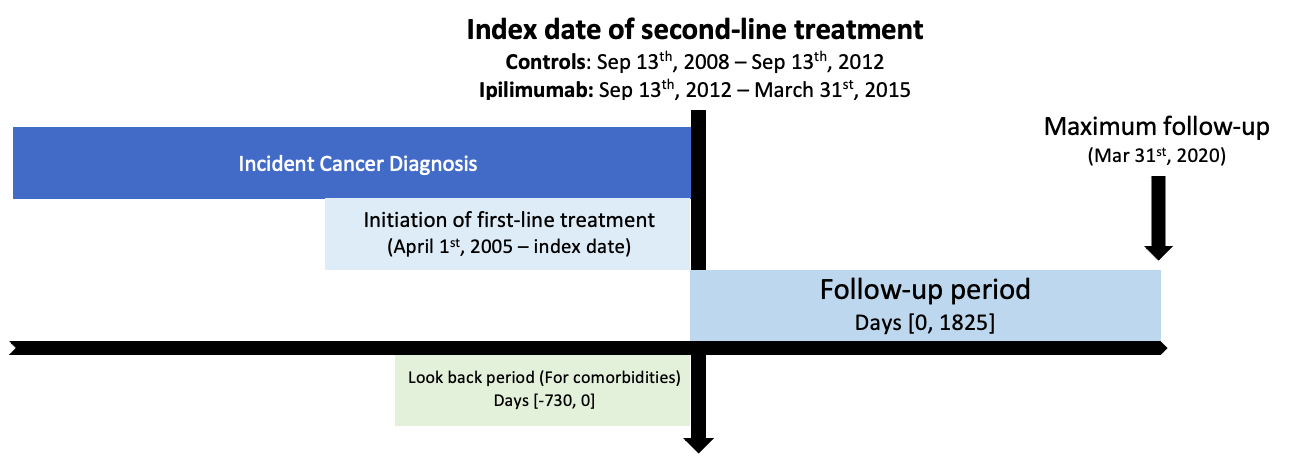


**Appendix B:** Supplementary methods for baseline covariates and costs

Death date, age, biological sex, and postal codes were obtained from the Registered Persons Database. Neighborhood income quintiles, health region of residence (Local Health Integrated Network, LHIN), and rurality status were calculated by linking individuals’ postal codes from to the Postal Code Conversion File and 2016 Statistics Canada data. Baseline systemic treatment and radiotherapy were obtained from the ALR. Patient comorbidity was quantified using a weighted average of the Adjusted Clinical Groups (ACG® System Aggregated Diagnosis Groups (ADG), Johns Hopkins ACG® System, Version 10), ADG score.^1^ Comorbidity measures were calculated using diagnoses codes obtained from hospital discharge records and physician claims records in the 2 years prior to the index date, excluding cancer diagnoses.

Costs associated with outpatient physician visits and laboratory tests in Ontario were estimated from the Physician Claims History Database of the Ontario Health Insurance Plan. Costs for emergency department visits and same‐day surgeries were estimated using the National Ambulatory Care Reporting System database. The costs of hospitalization, emergency department visits, and same‐day surgeries were estimated using the Resource Intensity Weight methodology developed by the Canadian Institute for Health Information.^2^ Prescription medication costs were obtained from the ODBP and NDFP, which are based on list prices. Costs associated with home care, continuing care, and long‐term care were estimated from the Ontario Home Care database and Continuing Care Reporting System. Costs were adjusted for inflation to 2018 Canadian dollars using the Statistics Canada Consumer Price Indices for healthcare services and medicinal and pharmaceutical products for Ontario.

References

1. Austin PC, Walraven C van. The mortality risk score and the ADG score: two points-based scoring systems for the Johns Hopkins Aggregated Diagnosis Groups to predict mortality in a general adult population cohort in Ontario, Canada. *Med Care*. 2011;49(10). https://journals.lww.com/lww-medicalcare/Fulltext/2011/10000/The_Mortality_Risk_Score_and_the_ADG_Score__Two.10.aspx

2. Canadian Institute for Health Information. The cost of hospital stays: Why costs vary. 2008. https://secure.cihi.ca/free_products/2008hospcosts_report_e.pdf

**Appendix C:** Kaplan-Meier survival estimates adjusted for IPTW

**Appendix D:** Data sources

|  | | **Data Source** |
| --- | --- | --- |
| **Cohort Characteristics** | | |
| Patient demographics | | Ontario Cancer Registry Registered Persons Database |
| Disease characteristics | | Ontario Cancer Registry |
| Treatment history | | Activity Level Reporting New Drug Funding Program CIHI Discharge Abstract Database |
| **Survival** | | |
| Recorded date of death | | Registered Persons Database |
| **Cost Category** | **Individual Cost Component** |  |
| Systemic therapy | | New Drug Funding Program Ontario Drug Benefit Activity Level Reporting |
| Ambulatory hospital care | Cancer clinic | National Ambulatory Care Reporting System |
|  | Hospital outpatient clinic | OHIP Physician and Laboratory Claims Database |
|  | Emergency department visit | National Ambulatory Care Reporting System |
|  | Day surgery | National Ambulatory Care Reporting System |
| Acute inpatient hospital care | | CIHI Discharge Abstract Database |
| Physician claims | | OHIP Physician and Laboratory Claims Database |
| Radiation | | Activity Level Reporting OHIP Physician Claims Database National Ambulatory Care Reporting Database Management Information System |
| Chronic and rehabilitation care | Complex continuing care | Continuing Care Reporting System |
|  | Long-term care | Continuing Care Reporting System Long-Term Care Database |
|  | Inpatient rehabilitation | National Rehabilitation Reporting System |
| Laboratory costs | | OHIP Physician and Laboratory Claims database |
| Home care services | | Home Care Database |
|  |  |  |
| *OHIP, Ontario Health Insurance Plan; CIHI, Canadian Institute for Health Information* | | |

**Appendix E**: Incremental net monetary benefit

**Table E1.** Estimates of incremental net monetary benefit and probability of cost-effectiveness by different WTP thresholds per LYG*

| **WTP Threshold**  **($/ LYG)** | **Per Patient** | | |
| --- | --- | --- | --- |
|  | **INMB estimate** | **95% CI** | **Probability of cost-effectiveness** |
| $50,000 | -$61,604 | -80,016, -43,190 | 0.0% |
| $100,000 | -$31,923 | -56,009, -7,838 | 1.7% |
| $150,000 | -$2,243 | -37,370, 32,885 | 50.6% |
| $200,000 | $27,438 | -20,526, 75,402 | 86.4% |
| $250,000 | $57,118 | -4,362, 118,599 | 95.9% |
| $300,000 | $86,799 | 11,487, 162,111 | 98.5% |
|  |  |  |  |
| *All costs have been rounded to the nearest dollar | | |  |
| *Cost and LY discounted at 1.5% | | |  |
| *WTP, willingness-to-pay; INMB, Incremental net monetary benefit; CI, confidence interval; LYG, life-years gained* | | | |

**Figure E1.** Estimates of incremental net monetary benefit by different WTP thresholds

*WTP, willingness-to-pay; LYG life-years gained*

**Appendix F**: CHEERS, RECORD-PE, and STaRT-RWE checklists

**CHEERS 2022 Checklist**

| **Topic** | **No.** | **Item** | **Location where item is reported** |
| --- | --- | --- | --- |
| **Title** |  |  |  |
|  | 1 | Identify the study as an economic evaluation and specify the interventions being compared. | Page 1 |
| **Abstract** |  |  |  |
|  | 2 | Provide a structured summary that highlights context, key methods, results, and alternative analyses. | Page 2,3 |
| **Introduction** |  |  |  |
| **Background and objectives** | 3 | Give the context for the study, the study question, and its practical relevance for decision making in policy or practice. | Page 4,5 |
| **Methods** |  |  |  |
| **Health economic analysis plan** | 4 | Indicate whether a health economic analysis plan was developed and where available. | n/a |
| **Study population** | 5 | Describe characteristics of the study population (such as age range, demographics, socioeconomic, or clinical characteristics). | Page 6,7 |
| **Setting and location** | 6 | Provide relevant contextual information that may influence findings. | Page 6,7 |
| **Comparators** | 7 | Describe the interventions or strategies being compared and why chosen. | Page 6 |
| **Perspective** | 8 | State the perspective(s) adopted by the study and why chosen. | Page 9 |
| **Time horizon** | 9 | State the time horizon for the study and why appropriate. | Page 8 |
| **Discount rate** | 10 | Report the discount rate(s) and reason chosen. | Page 9 |
| **Selection of outcomes** | 11 | Describe what outcomes were used as the measure(s) of benefit(s) and harm(s). | Page 8,9 |
| **Measurement of outcomes** | 12 | Describe how outcomes used to capture benefit(s) and harm(s) were measured. | Page 7-9 |
| **Valuation of outcomes** | 13 | Describe the population and methods used to measure and value outcomes. | Page 7-9 |
| **Measurement and valuation of resources and costs** | 14 | Describe how costs were valued. | Page 8,9 |
| **Currency, price date, and conversion** | 15 | Report the dates of the estimated resource quantities and unit costs, plus the currency and year of conversion. | Appendix B |
| **Rationale and description of model** | 16 | If modelling is used, describe in detail and why used. Report if the model is publicly available and where it can be accessed. | n/a |
| **Analytics and assumptions** | 17 | Describe any methods for analysing or statistically transforming data, any extrapolation methods, and approaches for validating any model used. | n/a |
| **Characterising heterogeneity** | 18 | Describe any methods used for estimating how the results of the study vary for subgroups. | n/a |
| **Characterising distributional effects** | 19 | Describe how impacts are distributed across different individuals or adjustments made to reflect priority populations. | n/a |
| **Characterising uncertainty** | 20 | Describe methods to characterise any sources of uncertainty in the analysis. | Page 7,9,10 |
| **Approach to engagement with patients and others affected by the study** | 21 | Describe any approaches to engage patients or service recipients, the general public, communities, or stakeholders (such as clinicians or payers) in the design of the study. | n/a |
| **Results** |  |  |  |
| **Study parameters** | 22 | Report all analytic inputs (such as values, ranges, references) including uncertainty or distributional assumptions. | Page 10, Table 1 |
| **Summary of main results** | 23 | Report the mean values for the main categories of costs and outcomes of interest and summarise them in the most appropriate overall measure. | Page 11,12  Table 2 |
| **Effect of uncertainty** | 24 | Describe how uncertainty about analytic judgments, inputs, or projections affect findings. Report the effect of choice of discount rate and time horizon, if applicable. | Page 10,11 |
| **Effect of engagement with patients and others affected by the study** | 25 | Report on any difference patient/service recipient, general public, community, or stakeholder involvement made to the approach or findings of the study | n/a |
| **Discussion** |  |  |  |
| **Study findings, limitations, generalisability, and current knowledge** | 26 | Report key findings, limitations, ethical or equity considerations not captured, and how these could affect patients, policy, or practice. | Page 12-14 |
| **Other relevant information** |  |  |  |
| **Source of funding** | 27 | Describe how the study was funded and any role of the funder in the identification, design, conduct, and reporting of the analysis | Page 14,15 |
| **Conflicts of interest** | 28 | Report authors conflicts of interest according to journal or International Committee of Medical Journal Editors requirements. | Page 15 |

*From:* Husereau D, Drummond M, Augustovski F, et al. Consolidated Health Economic Evaluation Reporting Standards 2022 (CHEERS 2022) Explanation and Elaboration: A Report of the ISPOR CHEERS II Good Practices Task Force. Value Health 2022;25. <doi:10.1016/j.jval.2021.10.008>

The RECORD statement for pharmacoepidemiology (RECORD-PE) checklist of items, extended from the STROBE and RECORD statements, which should be reported in non-interventional pharmacoepidemiological studies using routinely collected health data

| **Item No** | **STROBE items** | **RECORD items** | **RECORD-PE items** | **Page No** |
| --- | --- | --- | --- | --- |
| **Title and abstract** | | | | |
| 1 | (a) Indicate the study’s design with a commonly used term in the title or the abstract.  (b) Provide in the abstract an informative and balanced summary of what was done and what was found. | 1.1: The type of data used should be specified in the title or abstract. When possible, the name of the databases used should be included.  1.2: If applicable, the geographical region and timeframe within which the study took place should be reported in the title or abstract.  1.3: If linkage between databases was conducted for the study, this should be clearly stated in the title or abstract. | — | 1-3 |
| **Introduction** | | | | |
| Background rationale | | | | |
| 2 | Explain the scientific background and rationale for the investigation being reported. | — | — | 4,5 |
| Objectives | | | | |
| 3 | State specific objectives, including any prespecified hypotheses. | — | — | 5 |
| **Methods** | | | | |
| Study design | | | | |
| 4 | Present key elements of study design early in the paper. | — | 4.a: Include details of the specific study design (and its features) and report the use of multiple designs if used.  4.b: The use of a diagram(s) is recommended to illustrate key aspects of the study design(s), including exposure, washout, lag and observation periods, and covariate definitions as relevant. | 6,7, Appendix A |
| Setting | | | | |
| 5 | Describe the setting, locations, and relevant dates, including periods of recruitment, exposure, follow-up, and data collection. | — | — | 6,7 |
| Participants | | | | |
| 6 | (a) Cohort study—give the eligibility criteria, and the sources and methods of selection of participants. Describe methods of follow-up. Case-control study—give the eligibility criteria, and the sources and methods of case ascertainment and control selection. Give the rationale for the choice of cases and controls. Cross sectional study—give the eligibility criteria, and the sources and methods of selection of participants.  (b) Cohort study—for matched studies, give matching criteria and number of exposed and unexposed. Case-control study—for matched studies, give matching criteria and the number of controls per case. | 6.1: The methods of study population selection (such as codes or algorithms used to identify participants) should be listed in detail. If this is not possible, an explanation should be provided.  6.2: Any validation studies of the codes or algorithms used to select the population should be referenced. If validation was conducted for this study and not published elsewhere, detailed methods and results should be provided.  6.3: If the study involved linkage of databases, consider use of a flow diagram or other graphical display to demonstrate the data linkage process, including the number of individuals with linked data at each stage. | 6.1.a: Describe the study entry criteria and the order in which these criteria were applied to identify the study population. Specify whether only users with a specific indication were included and whether patients were allowed to enter the study population once or if multiple entries were permitted. See explanatory document for guidance related to matched designs. | 6,7 |
| Variables | | | | |
| 7 | Clearly define all outcomes, exposures, predictors, potential confounders, and effect modifiers. Give diagnostic criteria, if applicable. | 7.1: A complete list of codes and algorithms used to classify exposures, outcomes, confounders, and effect modifiers should be provided. If these cannot be reported, an explanation should be provided. | 7.1.a: Describe how the drug exposure definition was developed.  7.1.b: Specify the data sources from which drug exposure information for individuals was obtained.  7.1.c: Describe the time window(s) during which an individual is considered exposed to the drug(s). The rationale for selecting a particular time window should be provided. The extent of potential left truncation or left censoring should be specified.  7.1.d: Justify how events are attributed to current, prior, ever, or cumulative drug exposure.  7.1.e: When examining drug dose and risk attribution, describe how current, historical or time on therapy are considered.  7.1.f: Use of any comparator groups should be outlined and justified.  7.1.g: Outline the approach used to handle individuals with more than one relevant drug exposure during the study period. | 6-9 |
| Data sources/measurement | | | | |
| 8 | For each variable of interest, give sources of data and details of methods of assessment (measurement). Describe comparability of assessment methods if there is more than one group. | — | 8.a: Describe the healthcare system and mechanisms for generating the drug exposure records. Specify the care setting in which the drug(s) of interest was prescribed. | 6,7,9  Appendix B |
| Bias | | | | |
| 9 | Describe any efforts to address potential sources of bias. | — | — | 9,10 |
| Study size | | | | |
| 10 | Explain how the study size was arrived at. | — | — | 6,7  Appendix A |
| Quantitative variables | | | | |
| 11 | Explain how quantitative variables were handled in the analyses. If applicable, describe which groupings were chosen, and why. | — | — | 7-10 |
| Statistical methods | | | | |
| 12 | (a) Describe all statistical methods, including those used to control for confounding.  (b) Describe any methods used to examine subgroups and interactions.  (c) Explain how missing data were addressed.  (d) Cohort study—if applicable, explain how loss to follow-up was addressed. Case-control study—if applicable, explain how matching of cases and controls was addressed. Cross sectional study—if applicable, describe analytical methods taking account of sampling strategy.  (e) Describe any sensitivity analyses. | — | 12.1.a: Describe the methods used to evaluate whether the assumptions have been met.  12.1.b: Describe and justify the use of multiple designs, design features, or analytical approaches. | 7-10 |
| Data access and cleaning methods | | | | |
| 12 | — | 12.1: Authors should describe the extent to which the investigators had access to the database population used to create the study population.  12.2: Authors should provide information on the data cleaning methods used in the study. | — | 6 |
| Linkage | | | | |
| 12 | — | 12.3: State whether the study included person level, institutional level, or other data linkage across two or more databases. The methods of linkage and methods of linkage quality evaluation should be provided. | — | 6-9 Appendix B |
| **Results** | | | | |
| Participants | | | | |
| 13 | (a) Report the numbers of individuals at each stage of the study (eg, numbers potentially eligible, examined for eligibility, confirmed eligible, included in the study, completing follow-up, and analysed).  (b) Give reasons for non-participation at each stage.  (c) Consider use of a flow diagram. | 13.1: Describe in detail the selection of the individuals included in the study (that is, study population selection) including filtering based on data quality, data availability, and linkage. The selection of included individuals can be described in the text or by means of the study flow diagram. | — | 10, Appendix A |
| Descriptive data | | | | |
| 14 | (a) Give characteristics of study participants (eg, demographic, clinical, social) and information on exposures and potential confounders.  (b) Indicate the number of participants with missing data for each variable of interest.  (c) Cohort study—summarise follow-up time (eg, average and total amount). | — | — | 10, Table 1 |
| Outcome data | | | | |
| 15 | Cohort study—report numbers of outcome events or summary measures over time. Case-control study—report numbers in each exposure category, or summary measures of exposure. Cross sectional study—report numbers of outcome events or summary measures. | — | — | 10,11, Table 2 |
| Main results | | | | |
| 16 | (a) Give unadjusted estimates and, if applicable, confounder adjusted estimates and their precision (eg, 95% confidence intervals). Make clear which confounders were adjusted for and why they were included.  (b) Report category boundaries when continuous variables are categorised.  (c) If relevant, consider translating estimates of relative risk into absolute risk for a meaningful time period. | — | — | 10,11 Table 2, Figure 1A |
| Other analyses | | | | |
| 17 | Report other analyses done—eg, analyses of subgroups and interactions, and sensitivity analyses. | — | — | 11,  Table 3, Figure 2 |
| **Discussion** | | | | |
| Key results | | | | |
| 18 | Summarise key results with reference to study objectives. | — | — | 12 |
| Limitations | | | | |
| 19 | Discuss limitations of the study, taking into account sources of potential bias or imprecision. Discuss both direction and magnitude of any potential bias. | 19.1: Discuss the implications of using data that were not created or collected to answer the specific research question(s). Include discussion of misclassification bias, unmeasured confounding, missing data, and changing eligibility over time, as they pertain to the study being reported. | 19.1.a: Describe the degree to which the chosen database(s) adequately captures the drug exposure(s) of interest. | 13 |
| Interpretation | | | | |
| 20 | Give a cautious overall interpretation of results considering objectives, limitations, multiplicity of analyses, results from similar studies, and other relevant evidence. | — | 20.a: Discuss the potential for confounding by indication, contraindication or disease severity or selection bias (healthy adherer/sick stopper) as alternative explanations for the study findings when relevant. **[A: Original text indicated this item was RECORD (ie, not RECORD-PE)?]** | 13,14 |
| Generalisability | | | | |
| 21 | Discuss the generalisability (external validity) of the study results. | — | — | 12-14 |
| **Other information** | | | | |
| Funding | | | | |
| 22 | Give the source of funding and the role of the funders for the present study and, if applicable, for the original study on which the present article is based. | — | — | 14,15 |
| Accessibility of protocol, raw data, and programming code | | | | |
| 22 | — | 22.1: Authors should provide information on how to access any supplemental information such as the study protocol, raw data, or programming code. | — | 15 |

RECORD=reporting of studies conducted using observational routinely collected data; RECORD-PE=RECORD for pharmacoepidemiological research; STROBE=strengthening the reporting of observational studies in epidemiology.

*[REFERENCE: Langan SM, Schmidt S, Wing K, Ehrenstein V, Nicholls S, Filion K, Klungel O, Petersen I, Sorensen H, Guttmann A, Harron K, Hemkens L, Moher D, Schneeweiss S, Smeeth L, Sturkenboom M, von Elm E, Wang S, Benchimol EI. The REporting of studies Conducted using Observational Routinely-collected health Data (RECORD) Statement for Pharmacoepidemiology (RECORD-PE). *BMJ* 2018; 363: k3532.](https://www.bmj.com/content/363/bmj.k3532)

**Structured Template and Reporting Tool for Real World Evidence (STaRT-RWE) TABLE OF CONTENTS**

Table 1. Administrative Information

Table 2. Version History

Figure 1. Design Diagram

Table 3. Summary of Analytic Study Population

1. Meta-data about data source and software
2. Index Date (day 0) defining criterion
3. Inclusion Criteria
4. Exclusion Criteria
5. Predefined Covariates
6. Empirically Defined Covariates
7. Outcome
8. Follow up

Table 4. Analysis Specification

Table 5. Sensitivity Analyses

Table 6. Attrition Table

Table 7. Power and Sample Size Calculation

Table 8. Glossary of Terminology

Table 9. Abbreviations

**TABLE 1. ADMINISTRATIVE INFORMATION**

| Kelvin Chan | Principal Investigator | Sunnybrook Health Sciences Centre |
| --- | --- | --- |
| Wanrudee Isaranuwatchai | Co-Investigator | St. Michael’s Hospital |
| Wei Fang Dai | Co-Investigator | University of Toronto |
| Jaclyn Beca | Co-Investigator | Ontario Health |
| Ruth Croxford | Co-Investigator | Institute of Clinical Evaluative Sciences |
| Ines Menjak | Co-Investigator | Sunnybrook Health Sciences Centre |
| Teresa Petrella | Co-Investigator | Sunnybrook Health Sciences Centre |
| Timothy Hanna | Co-Investigator | Queen’s University |

151290

CIHR-CATALYST

151290

NA; study began before START-RWE became available

To evaluate the comparative cost-effectiveness of second-line ipilimumab compared to non-ipilimumab treatments (chemotherapy or targeted treatments) for patients with metastatic melanoma over a 5-year time horizon

ICES

NA

Comparative cost-effectiveness of second-line ipilimumab for metastatic melanoma: A real-world population-based cohort study of resource utilization in Ontario, Canada

**Instructions:**

Fill in the yellow highlighted sections where applicable.

**Primary:**

**Protocol registration:**

**Registration identifier**

**Registration date**

**Registration site**

**Protocol version:**

**Version number**

**Version date**

**Protocol Contributors:**

**Name**

**Role**

**Affiliation**

**Funding:**

**Grant identifier**

**Source**

**Data Use Agreement (DUA)**

**DUA identifier**

**Data provider**

**Data provider contact for data use agreements**

**Human Subjects/Ethics Approval**

**Submission identifier**

**Date of approval**

**Name of human subjects/ethics approval committee**

**Protocol Title:**

**Objective:**

*Include PICOTS (Patient, Intervention, Comparator, Outcome, Time-Horizon, Setting)*

**TABLE 2. VERSION HISTORY**

**Instructions:** Fill in the yellow highlighted sections to log changes and rationale for making changes made to the prior version of the protocol.

**Version date**

**Version number**

**Change log**

**Rationale for change**

# FIGURE 1. DESIGN DIAGRAM

**Instructions:** Create design diagram using the framework outlined in Schneeweiss et al. Graphical Depiction of Longitudinal Study Designs in Health Care Databases. Ann Intern Med. 2019;170:398–406. The diagram can be created using power point templates or other software program of choice. It is intended to be read from top to bottom, reflecting the order of operations to create an analytic cohort from a source longitudinal healthcare database. Temporality of assessment windows are clearly shown relative to the cohort entry (“index”) date, which is considered day 0. Bracketed number ranges denote the inclusive time windows for washout, inclusion/exclusion, and covariate assessment windows as well as follow up. Whether or not day 0 is included in an assessment window can also be visually distinguished by whether it overlaps the vertical arrow representing the cohort entry date

The diagram may include footnotes specifying the inclusion/exclusion criteria, covariates, and censoring criteria relevant to each assessment window.


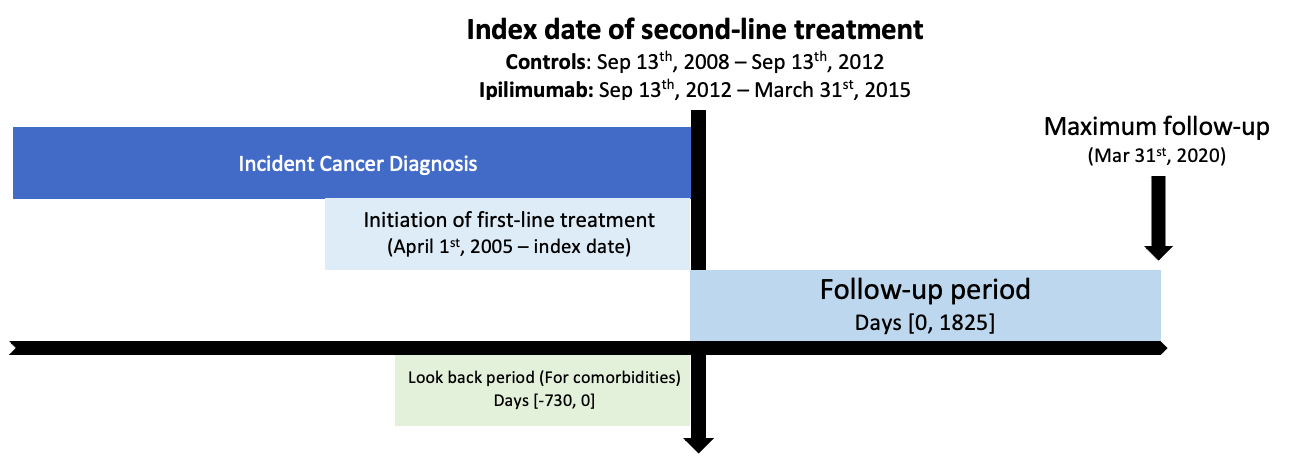


**TABLE 3. SUMMARY OF STUDY POPULATION PARAMETERS**

**Instructions:** Fill in the yellow highlighted sections. Example text included.

|  | **Data Source 1** | **Data Source 2** | **Data Source 3** | **Data Source 4** |
| --- | --- | --- | --- | --- |
| **Data source(s):** | Activity Level Reporting Database | New Drug Funding Program Database | Ontario Cancer Registry | Registered Persons Database |
| **Study period:** | Sep 13^th^, 2006 – Mar 31^st^, 2020 | Sep 13^th^, 2006 – Mar 31^st^, 2020 | Sep 13^th^, 2006 – Mar 31^st^, 2020 | Sep 13^th^, 2006 – Mar 31^st^, 2020 |
| **Eligible cohort entry period:** | Sep 13^th^, 2008 – Mar 31^st^, 2015 | Sep 13^th^, 2008 – Mar 31^st^, 2015 | Sep 13^th^, 2008 – Mar 31^st^, 2015 | Sep 13^th^, 2008 – Mar 31^st^, 2015 |
| **Data extraction**  **date/version:** |  |  |  |  |
| **Data sampling/extraction criteria:** | All enrollees in data source between Sep 13^th^, 2006 – Mar 31^st^, 2020 | All enrollees in data source between Sep 13^th^, 2006 – Mar 31^st^, 2020 | All enrollees in data source between Sep 13^th^, 2006 – Mar 31^st^, 2020 | All enrollees in data source between Sep 13^th^, 2006 – Mar 31^st^, 2020 |
| **Type(s) of data:** | Population-based database | Population-based database | Registry data | Population-based database |
| **Data linkage:** |  |  |  |  |
| **Data conversion:** |  |  |  |  |
| **Software to create study population:** |  |  |  |  |

|  | **Data Source 5** | **Data Source 6** | **Data Source 7** | **Data Source 8** |
| --- | --- | --- | --- | --- |
| **Data source(s):** | Canadian Institute for Health Information Discharge Abstract Database | Canadian Institute for Health Information National Ambulatory Reporting System | Ontario Health Insurance Plan Physician Claims Database | Ontario Drug Benefit Program |
| **Study period:** | Sep 13^th^, 2006 – Mar 31^st^, 2020 | Sep 13^th^, 2006 – Mar 31^st^, 2020 | Sep 13^th^, 2006 – Mar 31^st^, 2020 | Sep 13^th^, 2006 – Mar 31^st^, 2020 |
| **Eligible cohort entry period:** | Sep 13^th^, 2008 – Mar 31^st^, 2015 | Sep 13^th^, 2008 – Mar 31^st^, 2015 | Sep 13^th^, 2008 – Mar 31^st^, 2015 | Sep 13^th^, 2008 – Mar 31^st^, 2015 |
| **Data extraction date/version:** |  |  |  |  |
| **Data sampling/extraction criteria:** | All enrollees in data source between Sep 13^th^, 2006 – Mar 31^st^, 2020 | All enrollees in data source between Sep 13^th^, 2006 – Mar 31^st^, 2020 | All enrollees in data source between Sep 13^th^, 2006 – Mar 31^st^, 2020 | All enrollees in data source between Sep 13^th^, 2006 – Mar 31^st^, 2020 |
| **Type(s) of data:** | Population-based database | Population-based database | Population-based database | Population-based database |
| **Data linkage:** |  |  |  |  |
| **Data conversion:** |  |  |  |  |
| **Software to create study population:** |  |  |  |  |

|  | **Data Source 9** | **Data Source 10** | **Data Source 11** | **Data Source 12** |
| --- | --- | --- | --- | --- |
| **Data source(s):** | Ontario Home Care Database | Continuing Care Reporting System | National Rehabilitation Reporting System | Management Information System Database |
| **Study period:** | Sep 13^th^, 2006 – Mar 31^st^, 2020 | Sep 13^th^, 2006 – Mar 31^st^, 2020 | Sep 13^th^, 2006 – Mar 31^st^, 2020 | Sep 13^th^, 2006 – Mar 31^st^, 2020 |
| **Eligible cohort entry period:** | Sep 13^th^, 2008 – Mar 31^st^, 2015 | Sep 13^th^, 2008 – Mar 31^st^, 2015 | Sep 13^th^, 2008 – Mar 31^st^, 2015 | Sep 13^th^, 2008 – Mar 31^st^, 2015 |
| **Data extraction date/version:** |  |  |  |  |
| **Data sampling/extraction criteria:** | All enrollees in data source between Sep 13^th^, 2006 – Mar 31^st^, 2020 | All enrollees in data source between Sep 13^th^, 2006 – Mar 31^st^, 2020 | All enrollees in data source between Sep 13^th^, 2006 – Mar 31^st^, 2020 | All enrollees in data source between Sep 13^th^, 2006 – Mar 31^st^, 2020 |
| **Type(s) of data:** | Population-based database | Population-based database | Population-based database | Population-based database |
| **Data linkage:** |  |  |  |  |
| **Data conversion:** |  |  |  |  |
| **Software to create study population:** |  |  |  |  |

**A. Meta-data about data source and software**

This section records the calendar time range used to ascertain cohort entry (index date), as well as the calendar time range of data available for pre-index assessment windows and

post-index follow up (study period). The data source name and version are identified, as well as any sampling criteria applied (for example, the data cut only includes patients with a

diagnosis of diabetes). If there is data linkage involved, provide a citation or an appendix with description of the linkage (how, performance characteristics)

# TABLE 3. SUMMARY SPECIFICATION FOR ANALYTIC STUDY POPULATION

| Exposure | Date of incident administration of second-line ipilimumab for metastatic intent | Single | Incident | N/A | N/A | NDC | N/A | Ipilimumab | Yes | No | N/A |
| --- | --- | --- | --- | --- | --- | --- | --- | --- | --- | --- | --- |
| Comparator | Date of incident administration of non-ipilimumab treatment (chemotherapy or targeted treatments) for metastatic intent | Single | Incident | N/A | N/A | NDC | N/A | Non-ipilimumab (chemotherapy or targeted treatments) | Yes | No | N/A |

**Instructions:**

Fill in the yellow highlighted sections.

**B. Index Date (day 0) defining criterion**

**Study population**

**name(s)**

**Day 0 Description**

**Number of**

**entries**

**Type of**

**entry**

**Washout**

**window**

**Care**

**Setting¹**

**Code**

**Type**

**Diagnosis**

**position**

**²**

**Incident with respect to…**

**Pre-**

**specified**

**Varied for**

**sensitivity**

**Source of algorithm**

The criterion that define the date of entry to the cohort(s) is specified in this section. There should be one row for each unique definition of study population entry. If the study is descriptive, there may only be one row

filled out. An active comparator study may have 2 rows, one for the exposure of interest and one for the comparator.

Check the pre-specified box if the exclusion criterion was specified before beginning data analyses, check the varied for sensitivity box if it was modified as part of sensitivity analyses. Specify the source of algorithms to

define study entry criteria.

| **C. Inclusion Criteria**  Describe what the criterion is conceptually. Specify the order of application of the inclusion criteria is relative to selection of the index date (day 0) for study entry. For example, specify “after selection of index date” if you plan to 1) select the index date based on first time the study entry defining criterion is met in the study period, 2) then apply inclusion-exclusion criteria, 3) keep the selected index date for study entry if all inclusion-exclusion criteria are met. Alternatively, you can specify “before selection of index date” if you plan to 1) identify all potential index dates meeting the study entry criterion, 2) apply inclusion-exclusion criteria, 3) select one or more of the study entry dates that meet all inclusion-exclusion criteria. Define the assessment window relative to the index date, whether there are restrictions on care setting or diagnosis position in the algorithm to define each inclusion criterion and specify which study populations (defined in Table 3B) the criterion is applied to.  Defining “observable” patient time in the healthcare data source is almost always required as an inclusion criterion. When using administrative claims data, this can be measured with dates of enrollment in insurance coverage, with or without bridging of short gaps in enrollment. When using electronic health record data, defining observable patient time may require making some strong assumptions. For example, assuming that patient encounters are always observable, that patients are observable between the first and last recorded encounter in the record, that patients are observable for X days before and after any recorded encounter, etc. Alternatively, one could specify inclusion based on algorithms to measure “loyalty” to a healthcare provider or EHR system.  Check the pre-specified box if the exclusion criterion was specified before beginning data analyses, check the varied for sensitivity box if it was modified as part of sensitivity analyses. Specify the source of algorithms to define inclusion criteria. | | | |
| --- | --- | --- | --- |
| **Criterion Details Order of application** | **Assessment Care Code Type Diagnosis**  **window Settings¹ position²** | **Applied to study populations:** | **Pre- Varied for Source for algorithm specified sensitivity** |
| \| Observable time \| Melanoma diagnosis \| Before selection of index date \| [undefined, 0] \| N/A \| N/A \| N/A \| Exposure, Comparator \| Yes \| No \| N/A \| \| --- \| --- \| --- \| --- \| --- \| --- \| --- \| --- \| --- \| --- \| --- \| \| Observable time \| First-line treatment for metastatic melanoma \| Before selection of index date \| [April 1^st^, 2005, 0] \| N/A \| N/A \| N/A \| Exposure, Comparator \| Yes \| No \| N/A \| \| Received study drug of interest \| Second-line ipilimumab for metastatic melanoma \| Before selection of index date \| [0, 0] \| N/A \| N/A \| N/A \| Exposure \| Yes \| No \| N/A \| \| Received study drug of interest \| Second-line non-ipilimumab treatment for metastatic melanoma \| Before selection of index date \| [0, 0] \| N/A \| N/A \| N/A \| Comparator \| Yes \| No \| N/A \| | | | |
|  | | | |
|  | | | |

## TABLE 3. SUMMARY SPECIFICATION FOR ANALYTIC STUDY POPULATION

**Instructions:** Fill in the yellow highlighted sections.

| **D. Exclusion Criteria**  Describe what the criterion is conceptually. Specify the order of application of the exclusion criteria is relative to selection of the index date (day 0) for study entry. Define the assessment window relative to the index date, whether there are restrictions on care setting or diagnosis position in the algorithm to define each exclusion criterion and specify which study populations (defined in Table 3B) the criterion is applied to.  Check the pre-specified box if the exclusion criterion was specified before beginning data analyses, check the varied for sensitivity box if it was modified as part of sensitivity analyses. Specify the source of algorithms to define exclusion criteria. | | | |
| --- | --- | --- | --- |
| **Criterion Details Order of application** | **Assessment Care Code Type Diagnosis**  **window Settings¹ position²** | **Applied to study populations:** | **Pre-specified Varied for Source for algorithm sensitivity** |
|  | | | |
| \| Age <18 or >105 \| (Cohort entry date – date of birth) / 365 \| Before selection of index date \| [0, 0] \| N/A \| N/A \| N/A \| Exposure,  Comparator \| Yes \| No \| N/A \| \| --- \| --- \| --- \| --- \| --- \| --- \| --- \| --- \| --- \| --- \| --- \| \| Other cancer diagnosis \|  \| Before selection of index date \| [undefined, 0] \| N/A \| N/A \| N/A \| Exposure,  Comparator \| Yes \| No \| N/A \| \| Received ipilimumab in the first-line setting \|  \| Before selection of index date \| [undefined, 0] \| N/A \| N/A \| N/A \| Exposure,  Comparator \| Yes \| No \| N/A \| \| Received second-line clinical trial agents \|  \| Before selection of index date \| [undefined, 0] \| N/A \| N/A \| N/A \| Exposure,  Comparator \| Yes \| No \| N/A \| \| Received ipilimumab in combination with another drug \|  \| Before selection of index date \| [undefined, 0] \| N/A \| N/A \| N/A \| Exposure,  Comparator \| Yes \| No \| N/A \| | | | |
|  | | | |

**TABLE 3. SUMMARY SPECIFICATION FOR ANALYTIC STUDY POPULATION**

**Instructions:** Fill in the yellow highlighted sections.

| **E. Predefined Covariates**  Define the covariate conceptually, with accompanying details as necessary. Specify which planned analyses adjust for the covariate, and how it is specified in the analysis (e.g. continuous, categorical, binary). Define the assessment window relative to the index date (day 0), whether there are restrictions on care setting or diagnosis position in the algorithm, and which study populations defined in Table 3B the covariate is measured for. Specify the source of algorithms to define covariates.  Check the pre-specified box if the covariate was specified before beginning data analyses, check the varied for sensitivity box if it was modified as part of sensitivity analyses. Specify the source of algorithms to define covariates. | | |
| --- | --- | --- |
| **Characteristic Details Type of variable Assessment Care Code Diagnosis**  **window Settings¹ Type position²** | **Applied to study populations:** | **Pre- Varied for Source for algorithm**  **specified sensitivity** |
| \| Age at index \| (Index date – date of birth) \| Continuous \| [0, 0] \| N/A \| N/A \| N/A \| Exposure, Comparator \| Yes \| No \| N/A \| \| --- \| --- \| --- \| --- \| --- \| --- \| --- \| --- \| --- \| --- \| --- \| \| Sex \| Male, female, unknown \| Categorical \| [0, 0] \| N/A \| N/A \| N/A \| Exposure, Comparator \| Yes \| No \| N/A \| \| Income quintile \| Q1 to Q5 \| Categorical \| [0, 0] \| N/A \| N/A \| N/A \| Exposure, Comparator \| Yes \| No \| N/A \| \| Health region (LHIN) \| Region 1 to 14 \| Categorical \| [0, 0] \| N/A \| N/A \| N/A \| Exposure, Comparator \| Yes \| No \| N/A \| \| Rurality \| Urban, rural \| Binary \| [0, 0] \| N/A \| N/A \| N/A \| Exposure, Comparator \| Yes \| No \| N/A \| \| Morbidity score \|  \| Continuous \| [-730, 0] \| N/A \| N/A \| N/A \| Exposure, Comparator \| Yes \| No \| N/A \| \| Adjusted Diagnosis Groups \| 0-32 \| Continuous \| [-730, 0] \| N/A \| N/A \| N/A \| Exposure, Comparator \| Yes \| No \| N/A \| \| Charlson Comorbidity Index \| 0, 1+ \| Continuous \| [-730, 0] \| Hospit alizati on \| ICD-10 \| N/A \| Exposure, Comparator \| Yes \| No \| N/A \| \| Prior radiation (any) \| Yes, no \| Binary \| [undefined, 0] \| N/A \| N/A \| N/A \| Exposure, Comparator \| Yes \| No \| N/A \| \| Prior radiation of brain \| Yes, no \| Binary \| [undefined, 0] \| N/A \| N/A \| N/A \| Exposure, Comparator \| Yes \| No \| N/A \| \| Prior radiation other than brain \| Yes, no \| Binary \| [undefined, 0] \| N/A \| N/A \| N/A \| Exposure, Comparator \| Yes \| No \| N/A \| \| Prior resection (any) \| Yes, no \| Binary \| [undefined, 0] \| N/A \| N/A \| N/A \| Exposure, Comparator \| Yes \| No \| N/A \| \| Prior brain resection \| Yes, no \| Binary \| [undefined, 0] \| N/A \| N/A \| N/A \| Exposure, Comparator \| Yes \| No \| N/A \| \| Prior other resection \| Yes, no \| Binary \| [undefined, 0] \| N/A \| N/A \| N/A \| Exposure, Comparator \| Yes \| No \| N/A \| \| First-line treatment \| Chemotherapy, BRAF/MEK, non-ipilimumab immunotherapy, other \| Categorical \| [undefined, 0] \| N/A \| N/A \| N/A \| Exposure, Comparator \| Yes \| No \| N/A \| \| Time from diagnosis to index date \| (Index date – diagnosis date) \| Continuous \| [undefined, 0] \| N/A \| N/A \| N/A \| Exposure, Comparator \| Yes \| No \| N/A \| \| Time from end of first-line treatment to index date \| (Index date – first-line treatment end date) \| Continuous \| [undefined, 0] \| N/A \| N/A \| N/A \| Exposure, Comparator \| Yes \| No \| N/A \| \| Time from diagnosis to start of first-line treatment \| (First-line treatment date – diagnosis date) \| Continuous \| [undefined, 0] \| N/A \| N/A \| N/A \| Exposure, Comparator \| Yes \| No \| N/A \| | | |
|  | | |
|  | | |

# TABLE 3. SUMMARY SPECIFICATION FOR ANALYTIC STUDY POPULATION

**Instructions:** Fill in the yellow highlighted sections.

| **F. Empirically Defined Covariates**  Empirical identification of covariates to use in confounding control may not be relevant to all study populations or analyses, however if such methods are used, this section includes fields to describe what the algorithm for covariate identification is, as well as specification of the settings or parameters used to empirically identify covariates. In this section, specify the assessment window relative to the index date (day 0), which analyses adjust for empirically identified covariates, how the covariates are specified in a model, whether there are restrictions on care setting or diagnosis position, and which study populations (defined in section 3B) to measure the empirical covariates.  Check the pre-specified box if the empirical covariate selection parameters were specified before beginning data analyses, check the varied for sensitivity box if the parameters were modified as part of sensitivity analyses. Specify the source for the method and/or software used for empirically defined covariates. | | | |
| --- | --- | --- | --- |
| **Algorithm Type of variable** | **Assessment Care Code Type Diagnosis**  **window Settings¹ position²** | **Applied to study populations:** | **Pre- Varied for Source/code for specified sensitivity algorithm** |
|  | | | |
|  | | | |
|  | | | |

**TABLE 3. SUMMARY SPECIFICATION FOR ANALYTIC STUDY POPULATION**

**Instructions:** Fill in the yellow highlighted sections.

| **G. Outcome**  Define the outcome conceptually and whether it is the primary outcome of interest. Specify whether the type of outcome is incident (if so, there is a field to specify the washout window to define “incident” occurrences), prevalent or other. Specify whether there are restrictions on care setting or diagnosis position, and which groups or analyses the outcome is measured for. If there are measurement characteristics for the outcome algorithm (e.g. PPV, sensitivity, specificity) from publications, or from outcome validation within the study population (e.g., medical record review), provide this information.  Check the pre-specified box if the outcome parameters were specified before beginning data analyses, check the varied for sensitivity box if the parameters were modified as part of sensitivity analyses. Specify the source of algorithms to define outcomes. | | |
| --- | --- | --- |
| **Outcome name Outcome measurement characteristics Primary Type of Washout Care Code Diagnosis**  **outcome? outcome window Settings¹ Category position²** | **Applied to study populations:** | **Pre- Varied for Source of algorithm specified sensitivity** |
| \| Incremental cost-effectiveness ratio \| Cost and life-years gained or quality-adjusted life-years \| Yes \| Ratio \| N/A \| N/A \| N/A \| Primary \| Exposure, comparator \| Yes \| Yes \| N/A \| \| --- \| --- \| --- \| --- \| --- \| --- \| --- \| --- \| --- \| --- \| --- \| --- \| \| Incremental net monetary benefit \| Willingness-to-pay threshold, cost, life-years gained \| No \| Contin uous \| N/A \| N/A \| N/A \| Primary \| Exposure, comparator \| Yes \| No \| N/A \| | | |
|  | | |
|  | | |

# TABLE 3. SUMMARY SPECIFICATION FOR ANALYTIC STUDY POPULATION

| **Begins** | Day 1 |  |  |  |
| --- | --- | --- | --- | --- |
| **Ends** | **Select all that apply** | **Specify** | **Pre-specified** | **Varied for sensitivity** |
| **Date of Outcome** | No | N/A | N/A | N/A |
| **Date of Death** | Yes |  | Yes | No |
| **Date of Disenrollment** | Yes |  | Yes | No |
| **Day X following index date (specify date)** | Yes | Day 1825 (5 years maximum) | Yes | No |
| **End of exposure (specify operational details, e.g. stockpiling, algorithm, grace period** | No | N/A | N/A | N/A |
| **Date of add to/switch from exposure (specify algorithm)** | No | N/A | N/A | N/A |
| **Other (specify)** | No | N/A | N/A | N/A |

**Instructions:**

Fill in the yellow highlighted sections.

**H. Follow up**

Specify when follow up begins relative to the index date (day 0) and select each criterion that is used to end follow up.

Check the pre-specified box if the outcome parameters were specified before beginning data analyses, check the varied for sensitivity box if the parameters were

modified as part of sensitivity analyses.

Code algorithms for cohort entry date are provided in Appendix A, study entry criteria in Appendix B, covariates in Appendix C and D, outcomes in Appendix E (a * in

code algorithm indicates use of a wildcard)

All temporal windows anchored on study population entry date (Day 0) unless otherwise specified.

(

) represent open intervals that do not include the end points

] represent closed intervals that do include the end points

[

¹ Please enter all that apply. Valid entries: IP = inpatient, OP = outpatient, ED = emergency department, any, other, n/a = not applicable. See Appendix E for details on

how care setting is defined

Exposure, comparator

Incremental cost-effectiveness ratio

SAS 9.4: Proc Means, %getcost Macro at ICES

Outcome model: Ratio

(Incremental cost between cases vs controls) / (incremental benefit between cases vs controls)

Benefit is defined as both life-years gained or quality-adjusted life-years gained

Propensity score model: Logistic regression

Exposure =

Age at index +

Place of residence +

Comorbidity score +

Charlson score +

Number of aggregated diagnosis groups (ADGs) +

Collapsed ADGs +

Prior radiation to brain +

Prior radiation to other body parts +

Time from diagnosis to start of second-line treatment

Propensity score weighting

Missing Indicators

Exposure, comparator

Incremental net monetary benefit

SAS 9.4: Proc Means, %getcost Macro at ICES

Outcome model: Ratio

The net benefit (NB), $NB=\left( {effect}_{i} \right)-{cost}_{i}$, for each person (i) was calculated for the willingness-to-pay threshold (λ) beginning at $50,000

Propensity score model: Logistic regression

Exposure =

Age at index +

Place of residence +

Comorbidity score +

Charlson score +

Number of ADGs +

Collapsed ADGs +

Prior radiation to brain +

Prior radiation to other body parts +

Time from diagnosis to start of second-line treatment

Propensity score weighting

Assumption that if no relevant claims diagnoses/procedures are present, the condition is not present

Exposure increases cost and benefit relative to comparator

**TABLE 4 ANALYSIS SPECIFICATIONS**

**Instructions:**

Fill in the yellow highlighted sections.

**Primary**

**Secondary 1**

**Secondary 2**

**Hypothesis:**

**Study population(s)**

**Outcome:**

**Software:**

**Model(s):**

**Confounding adjustment method**

**Missing data methods**

d subgroup analyses.

Specify the study populations (defined in 3B) included in the analysis, the ou

tcome being evaluated, the software that is used, the type

of confounding adjustment, missing data methods an

Exposure increases cost and benefit relative to comparator

## TABLE 5 SENSITIVITY ANALYSES

To test if ICERs are sensitive to discount rate

To examine the impact on ICER

Discount rate, 0-3% (base 1.5%)

Price reduction of ipilimumab (from 0% to 100%)

**Instructions:**

Fill in the yellow highlighted sections.

**What is the parameter being varied?**

**(be clear what it is changing from)**

**Why? (What do you expect to learn?)**

**Strengths of the sensitivity analysis**

**compared to the primary?**

**Weaknesses of the sensitivity analysis**

**compared to the primary?**

**Sensitivity Analysis 1**

**Sensitivity Analysis 2**

## TABLE 5. ATTRITION TABLE

**Instructions:** Fill in the yellow highlighted sections. Show the number of patients remaining after applying each inclusion/exclusion criterion, sequentially.

|  | **Total Cohort** | | **Drug A** | | **Drug B** | |
| --- | --- | --- | --- | --- | --- | --- |
|  | **Excluded patients** | **Remaining patients** | **Excluded patients** | **Remaining patients** | **Excluded patients** | **Remaining patients** |
| All patients with melanoma who received second-line treatment for advanced melanoma after Sep 13^th^, 2008 | N/A | 728 |  |  |  |  |
| Age <18 years | 0 | 728 |  |  |  |  |
| Other cancer diagnosis | 0 | 728 |  |  |  |  |
| Non-ipilimumab treatment started on or after funding date | 257 | 471 |  |  |  |  |
| Received ipilimumab on or after funding date in combination with another drug | 47 | 424 |  |  |  |  |
| Received ipilimumab in the first-line setting | 6 | 418 |  |  |  |  |
| Received second-line clinical trial agents | 8 | 410 |  |  |  |  |
| Received ipilimumab prior to funding date | 81 | 329 |  |  |  |  |

Drug B initiators

N = 140

Drug A initiators

N = 189

# TABLE 7. POWER AND SAMPLE SIZE CALCULATION

**Instructions:** Fill in the yellow highlighted sections.

Specify the software used, what is being calculated (e.g. power, sample size, detectable difference), the population and statistical assumptions for the calculations. For each parameter assumption, specify the primary assumption and the range considered. Specify the sources used to select the estimated population parameters. The power or sample size calculations across the range of assumed parameter values may be displayed in tabular or visual form as needed.

The template contains assumptions to calculate power for a comparison of 2 proportions, however the entries for the population assumptions and statistical parameters should be modified to reflect those that are relevant for the calculation used by the investigators

Powerandsamplesize.com

Power to compare 2 proportions: 2-Sample, 2-Sided Equality

**Software:**

**Calculate:**

**Population assumptions**

**Primary**

**Range**

**Source for estimated parameters**

**Statistical parameters:**

# TABLE 8 GLOSSARY

## Term Definition

| Confounder | Variable other than the exposure of interest or outcome under investigation that is 1) associated with exposure, 2) a risk factor for the outcome, and 3) not on the causal pathway between the exposure and the outcome. A confounder can artificially inflate or reduce the magnitude of association between an exposure and outcome. |
| --- | --- |
| Covariates | Variables that are neither exposure nor outcome of interest, but are measured to describe a population or because they may be a confounder to account for in analyses |
| Data Extraction Date | The date when the data were extracted from the dynamic healthcare database |
| Days Supplied | Number of days supplied for a dispensed prescription |
| Eligible cohort entry period | Calendar time frame during which cohort entry dates are identified |
| Empirically defined covariates | Covariates that are not prespecified by the investigator. The selection of these covariates is based on applying algorithms to the data. The algorithms for covariate selection may be tuned by investigator specified parameters. |
| Observable Time | For insurance claims data, this may refer to periods of enrollment in medical and/or drug plans. For electronic health record databases, this may refer to algorithms designed to identify patients whose healthcare contacts are likely to be covered within the healthcare system. |
| Opbservable Time Gap | Maximum number of days allowed between two consecutive observable time windows to still be considered “continuously observable”. |
| Follow up window | The interval during which occurrence of the outcome of interest in the study population will be included in the analysis. |
| Grace Window | Number of days added to days supply to allow for non-adherence or account for the hypothesized biologic exposure risk window. Operationally, this could be defined as the number of extra days added to the end of a days supply to extend time counted as "exposed". This grace may bridge the gap between dispensations where the days supply dispensed does not cover all days until the refill. |
| Index Date | The date when subjects enter the study population (cohort entry date, outcome event date). It is defined based on events in the patient's longitudinal timeline, other windows are defined relative to the index date. |
| Assessment Window | Interval during which a patient is required to have evidence of a pre-existing condition (diagnosis/procedure/drug dispensing). May be used for washout of exposure or outcome, exclusion assessment or covariate assessment. |
| Predefined Covariates | Covariates that are prespecified and defined by the investigator in the protocol. |
| Principal Diagnosis | Diagnosis or condition established to be chiefly responsible for admission of the patient to the hospital. |
| Source Data Range | The calendar time range covered by a data source that is available |
| Stockpiling Algorithm | Algorithm defining how early refills are handled when determining length of exposure follow up |
| Study Period | Calendar time interval of data available for study, including pre-index date assessment windows and post-index follow up |
| Treatment Episode | Continuous period of exposure defined using by dispensation date + days supply and applying stockpiling algorithms and/or grace windows |
| Washout Window | Minimum number of days a patient is required to have no evidence of prior exposure and/or outcome |
| Wildcard | Symbol used to represent any single alphanumeric digit in code algorithms. For example 410.*1, where * is the wildcard. |

# ABBREVIATIONS

RX = drug prescription/dispensation

DX = diagnosis

PX = procedure

LB = laboratory

NDC = national drug code

ICD-9-CM = International Classification of Diseases - Clinical Modification 9th revision (2012)

ICD-10-CM = ICD-CM 10th revision

ICD-11-CM = ICD-CM 11th revision

CPT = Current Procedural Terminology

HCPCS = Healthcare Common Procedure Coding System

LOINC = Logical Observation Identifiers Names and Codes

IP = inpatient

OP = outpatient

ED = emergency department

N/A = not applicable
